# Supplementary material for: Reproduction ratio and growth rates: Measures for an unfolding pandemic
Source: PLoS One. 2020 Jul 23;15(7):e0236620. doi: 10.1371/journal.pone.0236620 (PMC7377402; doi:10.1371/journal.pone.0236620)
Supplement: Attachment — Submitted filename: Review-PONE-D-20-14985.pdf [file pone.0236620.s001.pdf]

Review on the manuscript PONE-D-20-14985
Reproduction ratio and growth rates: measures for an unfolding pandemic

The presented paper provides an interesting and valuable contribution to the current issue of estimating the current growth rates for the COVID–19 disease. Based on a variant of a SIR–type model, the authors estimate both the current growth rate and the reproduction number based on data for the Basque country. However, the presented approach can be directly transferred to other data sets. The results can serve as decision support for public authorities and health agencies.

The mathematical content of the paper is sound and applies rather standard techniques to a problem of high importance. However the manuscript itself seems to be in an early stage of writing requiring careful reading and elimination of double words and incomplete sentences.

I recommend the manuscript for publication after a minor revision.

- Abstract, line 12: double "the".
- Page 2, line 6: Uses as ~~a~~ public health tools ...
- Page 2, line 18: double "to"
- Page 2, line 27: double "momentary"
- Page 2, line 43–47: The sentence starting with "Although the absolute ..." is not understandable to me.
- Page 2, line 48: "care ~~unites~~units (ICU) ..."
- Page 3, line 71ff: What do you mean by positive tested patients in "yellow"? Does this refer to the later figures? I just have them in black & white...
- Page 3, Section 0.1 Please explain the variables SHARUCD of the model.
- Page 4, Eqn.(3) In the equation for C_H I assume a term $(1 - \nu)$ is missing
- Page 4, line 105: sever should read as "severe"
- Page 4, line 108ff: The explanation about ϱ appears twice in the text. Seemingly left over from a draft version of the paper.
- Page 5, Table 1: The authors use a value of 1.6 for the ratio ϕ of asymptotic infections compared to severe/hospitalized cases. Can you comment on this. I would assume here a significantly higher value since severe hospitalized cases are under strict quarantine and hence will not contribute significantly to the force of infection.
- Page 5, Table 1: Please comment on the choice of the given parameters or refer to sources where to find those values.

- Page 5, Table 1: Please use the same symbol for the letter "rho". On page 4 it is ϱ and on Page 5 it is ρ.
- Page 7, line 177: The cotangent does not show up here, so I guess "cot" is a typo.
- Page 7, line 188f: This seems to be again a left over from a draft version...
- Page 7, line 190ff: Please use `\ln` for the logarithm.
- Page 8, Figure 1: Do the black dots indicate to reported data? Please comment.
- Page 9, Figures 2 & 3: In the last line of the captions, it should read as "...shown in b) and d) for $\gamma^{-1} = \ldots$"
