# Supplementary material for: Reproduction ratio and growth rates: Measures for an unfolding pandemic
Source: PLoS One. 2020 Jul 23;15(7):e0236620. doi: 10.1371/journal.pone.0236620 (PMC7377402; doi:10.1371/journal.pone.0236620)
Supplement: Attachment — Submitted filename: ResponsetoReviewers.pdf [file pone.0236620.s002.pdf]

Dear Editor and Reviewer of PloS One Journal,

Thank you for your comments and for allowing us to revise our manuscript. In the revised version we addressed your comments, criticisms and suggestions.

We thank the referee for carefully reading our paper and for the polite tone of making comments. We really appreciated that.

Please, find below more details about your questions. In black are the comments from the reviewer, in blue are the answers from the authors. Changes can be found along the revised manuscript.

Reviewer(s)' Comments to Author:

The presented paper provides an interesting and valuable contribution to the current issue of estimating the current growth rates for the COVID–19 disease. Based on a variant of a SIR–type model, the authors estimate both the current growth rate and the reproduction number based on data for the Basque country. However, the presented approach can be directly transferred to other data sets. The results can serve as decision support for public authorities and health agencies.
The mathematical content of the paper is sound and applies rather standard techniques to a problem of high importance. However the manuscript itself seems to be in an early stage of writing requiring careful reading and elimination of double words and incomplete sentences.
I recommend the manuscript for publication after a minor revision.

• Abstract, line 12: double "the". - corrected
• Page 2, line 6: Uses as a public health tools . . . - corrected
• Page 2, line 18: double "to" - corrected
• Page 2, line 27: double "momentary" - corrected
• Page 2, line 43–47: The sentence starting with "Although the absolute . . . " is not understandable to me.

We have rephrased this sentence. It now reads:

"Although the absolute value of r(t) can vary given the modeling assumptions considered during its estimation, it is advised to rather look at the threshold behavior, using the growth rates primarily as it is independent of modeling uncertainties and clearly indicates if the outbreak is under control or not when estimations are below or above threshold."

• Page 2, line 48: "care unitesunits (ICU) . . . "- corrected
• Page 3, line 71ff: What do you mean by positive tested patients in "yellow"? Does this refer to the later figures? I just have them in black & white. . .

Yes. The color code was in the wrong place. It is now corrected. Empirical data are plotted as black dots and stochastic simulations in color. That is now referenced in the figure caption.

• Page 3, Section 0.1 Please explain the variables SHARUCD of the model.

The variables are now explained. It now reads:

“We use SHARUCD-type models, an extension of the well known simple SIR (susceptible-infected-recovered) model, with susceptible class S, infected class partitioned into severe infections prone to hospitalization (H) and mild, sub-clinical or asymptomatic infections (A). For severe infections prone to hospitalization, we assume that individuals could either recover R, be admitted to the ICU facilities U or oreventually decease into class D. Since we investigate the cumulative data on the infection classes (and not prevalence), we also include classes C to count cumulatively the new cases for “hospitalized” C_H , “asymptomatic” C_A , recovered C_R and ICU patients C_U . The deceased cases are automatically collecting cumulative cases, since there is no exit transition form the deceased class D.”

• Page 4, Eqn.(3) In the equation for C H I assume a term (1 − ν) is missing

- corrected. We are glad you understood the system well. Thanks for spotting this typo!

• Page 4, line 105: sever
should read as “severe”- corrected.

::::
• Page 4, line 108ff: The explanation about % appears twice in the text. Seemingly left over from a draft version of the paper.

Indeed. Corrected. Thanks!

• Page 5, Table 1: The authors use a value of 1.6 for the ratio φ of asymptotic infections compared to severe/hospitalized cases. Can you comment on this. I would assume here a significantly higher value since severe hospitalized cases are under strict quarantine and hence will not contribute significantly to the force of infection.

We have included the following paragraphs:

“Model parameters were estimated and fixed as the model is able to describe the disease incidence during the exponential phase of the outbreak. Parameter values were kept close to the ones found in the literature [12–17], used as baseline for initial values and then refined using the available data via the analysis of the likelihood functions [11]. The ratio of mild/asymptomatic infection contribution to the force of infection is estimated to be higher than the contribution of severe hospitalized cases since they would be more mobile and therefore able to transmit the disease while severe cases would be under strict quarantine once detected and admitted to a hospital. We model the effect of transmission of the overall hospitalized individuals and before the hospital admission is notified, symptomatic individuals would contribute significantly to the force of infection. The same assumption is valid for the mild/asymptomatic individuals.

In good agreement, the refined model can describe the hospitalizations, the ICU admissions and the deceased cases, well matched within the median of the 200 stochastic realizations (see Fig. 1). The cumulative incidence for all positive cases follows the higher stochastic realizations range and the deviation observed between model simulations and data can be explained by the increasing testing capacities since March 22, 2020, followed by the introduction of rapid tests (blue dots), mostly used as screening tool in nursing homes and public health workers."

Moreover, Figure 1 was updated with a large font size. In Fig. 1 a) we have added, for reference, data from "Data set B", i.e, notified positive cases with both diagnostic tests combined, RT-PCR and rapid tests.

• Page 5, Table 1: Please comment on the choice of the given parameters or refer to sources where to find those values.

Besides the paragraph above, we have also cited ref 11 for further details on parameter estimation and likelihood function analysis.

• Page 5, Table 1: Please use the same symbol for the letter "rho". On page 4 it is %
and on Page 5 it is ρ.- corrected.
• Page 7, line 177: The cotangent does not show up here, so I guess "cot" is a typo.- corrected.
• Page 7, line 188f: This seems to be again a left over from a draft version… Indeed. Corrected. Thanks!
• Page 7, line 190ff: Please use \ln for the logarithm.- corrected.
• Page 8, Figure 1: Do the black dots indicate to reported data? Please comment.

Yes. We have referenced that in the caption of Figure 1. It now reads

"Ensemble of 200 stochastic realizations of the SHARUCD-type model. Mean of the stochastic realizations is shown with a blue line. a) Cumulative tested positive cases $I_{cum}(t)$. Data set From April 6, 2020, we note an increase of reported positive cases as the testing capacities were increasing. In b) cumulative hospitalized cases $C_H (t)$, c) cumulative ICU admission $C_U (t)$, d) cumulative deceased cases $D(t)$. For reference, ``Data set A'' is plotted as black dots in a-c) and red dots in d). ``Data set B'' is plotted as blue dots in a). Empirical data was provided by the Basque Health Department and the Basque Health Service (Osakidetza)"

• Page 9, Figures 2 & 3: In the last line of the captions, it should read as ". . . shown in b) and d) for γ −1 =. . . " - corrected.

We believe that the revised manuscript follows a consistent and self explained format where we prepare the reader for analysis that will be performed along the manuscript. With all the corrections and explanations, we look forward to your positive feedback.

With my best regards,

Maira
